# Herb-Drug Interaction of *Epimedium sagittatum* (Sieb. et Zucc.) Maxim Extract on the Pharmacokinetics of Sildenafil in Rats

**DOI:** 10.3390/molecules18067323

**Published:** 2013-06-21

**Authors:** Thomas Y. Hsueh, Yu-Tse Wu, Lie-Chwen Lin, Allen W. Chiu, Chi-Hung Lin, Tung-Hu Tsai

**Affiliations:** 1 Institute of Traditional Medicine, School of Medicine, National Yang-Ming University, Taipei 11221, Taiwan; E-Mails: yjhsueh@gmail.com (T.Y.H.); joseph.wu2011@gmail.com (Y.-T.W.); 2 Department of Urology, School of Medicine, National Yang-Ming University, Taipei 11221, Taiwan; E-Mail: whchiu@ym.edu.tw (A.W.C.); 3 Department of Education and Research, Taipei City Hospital, Taipei 10341, Taiwan; 4 National Research Institute of Chinese Medicine, Taipei 11221, Taiwan; E-Mail: lclin@nricm.edu.tw (L.-C.L.); 5 Institute of Microbiology and Immunology, National Yang-Ming University, Taipei 11221, Taiwan; E-Mail: linch@ym.edu.tw (C.-H.L.); 6 Graduate Institute of Acupuncture Science, China Medical University, Taichung 40402, Taiwan

**Keywords:** sildenafil, *Epimedium sagittatum* extract, herb-drug interaction, Traditional Chinese Medicine

## Abstract

*Epimedium sagittatum* (Sieb. et Zucc.) Maxim is one of the herbs used to treat erectile dysfunction in Traditional Chinese Medicine. Sildenafil is a phosphodiesterase 5 inhibitor used to treat erectile dysfunction in Western Medicine. This study evaluates the herbal-drug interaction of *Epimedium sagittatum* extract on the pharmacokinetics of sildenafil in rats by ultra-performance liquid chromatography. The rat plasma was sampled from each anesthetized rat after pretreatment with 3-days *Epimedium sagittatum* extract (1/2 g/kg/day) and intravenous injection with sildenafil (10/30 mg/kg). The pharmacokinetic data demonstrate that the area under the concentration-time curve (AUC) of sildenafil (10 mg/kg) was significantly decreased in groups that received a high dose of *Epimedium sagittatum* extract. In conclusion, the study demonstrates that there was significant herb-drug interaction of *Epimedium sagittatum* extract on the pharmacokinetics of sildenafil at low and high daily doses, suggesting co-administration use of *Epimedium sagittatum* extract and sildenafil in clinical practice should be prevented due to possible herb-drug interactions.

## 1. Introduction

Erectile dysfunction is an age-related problem with a prevalence rate of 17.7% in men older than 40, and it increases with age and number of comorbidities [1]. The inability to maintain erectile function results in anxiety, loss of self-esteem and relationship strains [2,3]. It is not surprising erectile dysfunction affects interpersonal relationships between partners, resulting in diminished psychosocial functioning in men with erectile dysfunction. Treatment of erectile dysfunction leads to improved quality of life in both the sexual and emotional aspects [4,5,6]. Since 1998, sildenafil, one of the phosphodiesterase-5 inhibitors, which acts by regulating the blood flow of penis via inhibition of cGMP specific phosphodiesterase 5 in the corpus cavernosum, was considered the treatment of choice for erectile dysfunction, with a response rate ranging from 68–69% [7]. Sildenafil was absorbed and acted within 30 min to 150 min while a mean half-life of approximately 240 min over the dose range of 25 mg to 200 mg. The mean absolute oral bioavailability is 41% after single oral intake of sildenafil and food imposes a small reduction in the rate and extent of systemic exposure [8]. Sildenafil is metabolized by liver microsomes and CYP3A4 and excreted through either the urinary tract or gastrointestinal tract [9,10]. There are some possible side effects mentioned in the literature, such as hot flushes, blue tinged vision, headache, and dizziness. Despite this, it remains the treatment of choice for men with mild to moderate erectile dysfunction [11]. 

*Epimedium sagittatum* is one of the Herba Epimedii species and is distributed from middle Asia to southeast China [11]. The major components of *Epimedium sagittatum* are icariin, epimedium B and epimedin C. It is reported to have anti-inflammatory, anti-proliferative, and anti-tumor effects [12,13,14]. It is also reported to have potential effects on the management of erectile dysfunction [15,16,17]. *Epimedium sagittatum* has been used to treat male erectile dysfunction in Traditional Chinese Medicine for many centuries. The main functions of *Epimedium sagittatum* in ancient Chinese books focused on the nourishment of kidney viscera and reinforcement of ‘yang’, resulting in the restoration of erectile function in males [18]. Although *Epimedium sagittatum* is reported to have multiple treatment effects in Traditional Chinese Medicine, the pharmacokinetic profile data in the literature is limited, and is mainly focused on icariin, the major purified product from the *Epimedium sagittatum* species [19,20]. Previous studies have indicated epimedii herba extract modulates liver metabolic enzyme activities, such as CYP1A2, CYP3A4 and CYP2E1, which are the main isoforms of cytochrome P450, and total flavonoids may be responsible for the inhibition the metabolism of sildenafil [13,21]. Our recent work has demonstrated biliary excretion is the major elimination pathway for icariin, while P-glycoprotein might play a role in the metabolism of icariin in rats [22]. 

The cultural differences of Asian and Western countries result in different attitudes and willingness to receive treatment for erectile dysfunction. In Asian countries, men with erectile dysfunction are primarily seeking alternative/Traditional Chinese Medicine instead of/along with Western Medicine [23]. Hence, the combination use of Western Medicine and Traditional Chinese Medicine is a common phenomenon for Asian [24]. Since *Epimedium sagittatum* has been used to treat erectile dysfunction in Traditional Chinese Medicine for a long time, the use of oral phosphodiesterase 5 inhibitors and *Epimedium sagittatum* for managing erectile dysfunction has probably been in practice since the initial use of phosphodiesterase 5 inhibitors for Asians. In 2007, Zhang reported the switching phenomenon between Traditional Chinese Medicine and sildenafil among impotence patients became common after the application of sildenafil in China [24]. They reported that switching back and forth between sildenafil and Traditional Chinese Medicine (mainly herba epimedii) represents not only two types of medications, but also revolves around the sexual desire and the cultivation of life in China. Hence, the investigation of herbal drug effect between sildenafil and herba epimedii deserves further investigation so as to understand the possible safety issues regarding the combination use of sildenafil and herba epimedii. 

According to a survey of the PubMed database, the pharmacokinetic interactions for the combined use of *Epimedium sagittatum* and sildenafil have not been reported in the literature. To understand the possible herbal drug interaction between *Epimedium sagittatum* and phosphodiesterase 5 inhibitors, the pharmacokinetic interaction of the *Epimedium sagittatum* species and sildenafil should be understood. The aim of this study is to investigate the herbal drug pharmacokinetic interaction of *Epimedium sagittatum* extract and sildenafil in rats. To explore the dose related interaction of the herbal extract and sildenafil, the experimental animals were divided into six groups to compare sildenafil administration alone, and a low (1 g/kg/day) or high (2 g/kg/day) dose of *Epimedium sagittatum* extract was given for three consecutive days of pretreatment, and on 4^th^ day a low (10 mg/kg) or high (30 mg/kg) dose of sildenafil was given. 

## 2. Results and Discussion

### 2.1. Analytical Method Validation for Sildenafil

**Figure 1:**
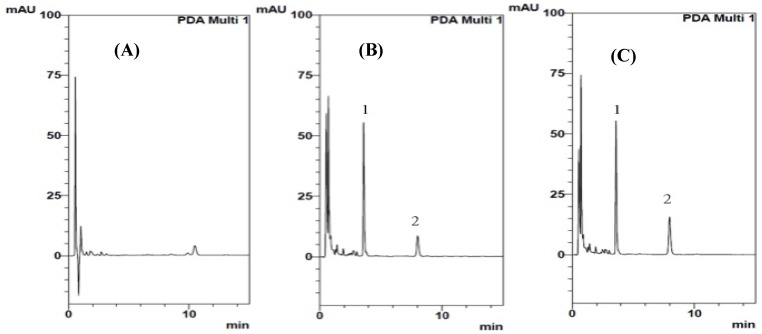
HPLC chromatograms of intravenous sildenafil after oral administration of the *Epimedium sagittatum* extract. (**A**) blank plasma, (**B**) blank plasma spiked with sildenafil (1 µg/mL), (**C**) plasma sample collected at 15 min after treatment of epimedii extract 2 h before sildenafil administration (10 mg/kg). 1: Internal Standard (*p*-hydrobenzoate), 2: sildenafil.

The developed analytical method was used to determine the sildenafil level in rat plasma and the chromatogram is shown in Figure 1. The retention time for the internal standard (*p*-hydrobenzoate) and sildenafil were 3.7 min and 8 min, respectively. There was no interference during the retention time of 6–10 min in the blank plasma dialysate. The accuracy was calculated by the nominal concentration (Cnom) and the mean value of the observed concentrations (Cobs) as follows: accuracy (%) = (Cobs/Cnom) × 100. The precision, relative standard deviation (RSD), was estimated from the observed concentrations as follows: RSD (%) = (standard deviation (SD)/Cobs) × 100. Table 1 showed the analytical precision and accuracy of sildenafil in rat plasma. The calibration curve for sildenafil had good linearity (R^2^ = 0.996) over a range of 0.05 μg/mL to 10 μg/mL. The inter-day and intra-day assay had relative standard deviation values less than 8.1%, while the accuracy rate ranged from 97.8% to 115.6%. These stability results showed no significant difference between the initial and tested concentrations. 

**Table 1:** Inter-day and intra-day assay of sildenafil in rat plasma.

**Nominal concentration (μg/mL)**	**Observed concentration (μg/mL)**	RSD (%) *	Accuracy (%) **
Interday			
0.05	0.06 ± 0.005	8.1	115.6
0.1	0.10 ± 0.006	6.1	98.2
0.5	0.50 ± 0.027	5.3	100.5
1	0.99 ± 0.061	6.1	99.5
5	4.99 ± 0.240	4.8	99.9
10	10.00 ± 0.563	5.6	100.0
Intraday			
0.05	0.05 ± 0.002	4.4	103.9
0.1	0.10 ± 0.004	4.6	97.9
0.5	0.50 ± 0.011	2.2	100.0
1	0.98 ± 0.024	2.4	97.8
5	5.04 ± 0.074	1.5	100.8
10	9.98 ± 0.264	2.6	99.8

Data are expressed as mean ± S. D. (n = 6). * The relative standard deviation (RSD) was estimated from the observed concentrations as follows: RSD (%) = (standard deviation (SD)/Cobs) × 100. ** The accuracy was calculated by the nominal concentration (Cnom) and the mean value of the observed concentrations (Cobs) : accuracy (%) = (Cobs/Cnom) × 100.

### 2.2. Pharmacokinetics of Sildenafil in Low Dose Epimedium sagittatum Extract (1 g/kg/day, p.o.)

**Figure 2:**
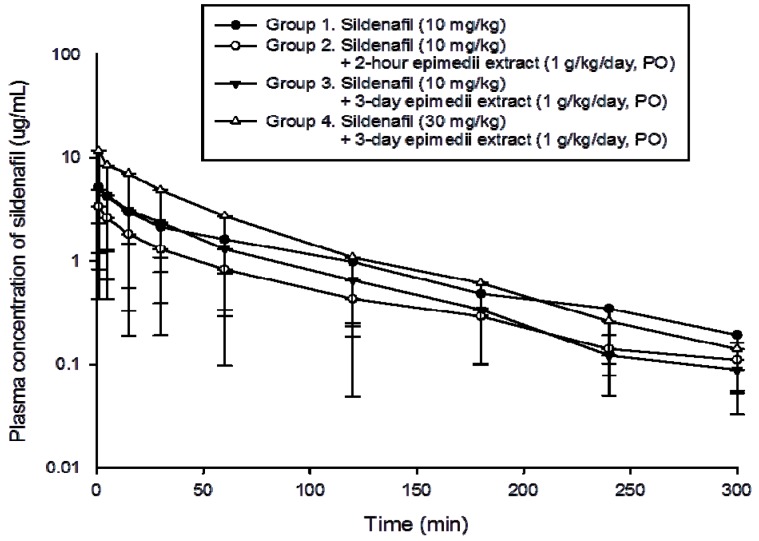
Plasma concentration-time profile of intravenous sildenafil in the blood of group 1: sildenafil alone (10 mg/kg); group 2: Low dose *Epimedium sagittatum* (Sieb. et Zucc.) Maxim extract (1 g/kg) 2 h pretreated + sildenafil (10 mg/kg), group 3: low dose *Epimedium sagittatum* (Sieb. et Zucc.) Maxim extract (1 g/kg/day) for 3 consecutive days and on day 4 + low dose sildenafil (10 mg/kg); group 4: low dose *Epimedium sagittatum* (Sieb. et Zucc.) Maxim extract (1 g/kg/day) for 3 consecutive days and on day 4 + high dose sildenafil (30 mg/kg). Data are presented as expressed as mean ± standard error of the mean (S.E.), n = 6 for each group.

The dosage regimens of *Epimedium sagittatum* extract and sildenafil are listed in Table 2. Figure 2 showed the concentration-time profiles in the groups received the 1 g/kg *Epimedium sagittatum* extract pretreatment (groups 1–4). 

**Table 2:** The dosage regimens of *Epimedium sagittatum* (Sieb. et Zucc.) Maxim extract and sildenafil administration ^a^.

**Treatment group**	**Dose of oral Epimedium sagittatum (Sieb. et Zucc.) Maxim extract ^b^**	**No. of herbal doses**	**Sildenafil dose**
1 ^c^	None	0	10 mg/kg
2	1 g/kg/day, 2h before sildenafil administration	1	10 mg/kg
3	1 g/kg/day	3	10 mg/kg
4	1 g/kg/day	3	30 mg/kg
5	2 g/kg/day	3	10 mg/kg
6	2 g/kg/day	3	30 mg/kg

^a^ Each group contained six rats. ^b^*Epimedium sagittatum* (Sieb. et Zucc.) Maxim extract was suspended in 2% carboxymethyl cellulose (1 g/mL). ^c^ Group 1 received vehicle (2% carboxymethyl cellulose, 1 g/mL) for 3 days as the control group

The half-life (t_1/2_) and Cl revealed no significant difference in groups 1, 2, 3 and 4 (Table 3). Since the initial concentration relied on the initial dosage of sildenafil, the C_0_ showed statistical significance between groups 1 and 4 (5.6 *versus* 11.6, *p* < 0.001), between groups 2 and 4 (5.3 *versus* 11.6, *p* < 0.001) and between groups 3 and 4 (3.4 *versus* 11.6, *p* < 0.001), but there was no statistical significance between groups 2 and 3 (3.4 *versus* 5.3, *p* = 0.24).

The AUC data showed statistically significant differences between groups 1 and 4 (348 *versus* 536, *p* < 0.001) and between groups 1 and 2 (348 *versus* 183, *p* = 0.001), while similar results were found between groups 3 and 4 (296 *versus* 536, *p* < 0.001) and between groups 2 and 3 (183 *versus* 296, *p* = 0.034). The AUC data showed statistically significant differences between group 1 and 2 and between groups 2 and 3 possibly suggest the herb-drug interaction between *Epimedium sagittatum* extract and sildenafil. The volume of distribution at steady state (V_ss_) showed significant results between groups 2 and 3 (5400 *versus* 2460, *p* = 0.037). 

**Table 3:** Pharmacokinetic parameters of sildenafil in various groups.

**Parameters**	**t_1/2_ (min)**	**C_0_ (μg/mL)**	**AUC (min μg/mL)**	**Cl (mL/min/kg)**	**V_ss_ (L/kg)**
Group 1	80 ± 34	5.6 ± 0.9	348 ± 44	27 ± 3.6	2710 ± 627
Group 2	84 ± 17	3.4 ± 0.8	183 ± 15 *	55 ± 5.0	5400 ± 588
Group 3	54 ± 3.8	5.3 ± 0.9	296 ± 24 ^†^	33 ± 2.8	2460 ± 98 ^†^
Group 4	61 ± 8.5	11.6 ± 2.3 *^,†,‡^	536 ± 86 *^,†,‡^	57 ± 8.9	3790 ± 552

Group 1, sildenafil alone (10 mg/kg); group 2, low dose *Epimedium sagittatum* extract (1 g/kg) 2 hr pretreated + low dose sildenafil (10 mg/kg); group 3, low dose *Epimedium sagittatum* extract (1 g/kg/day) for 3 consecutive days and on day 4^th^ + low dose sildenafil (10 mg/kg); group 4, low dose *Epimedium sagittatum* extract (1 g/kg/day) for 3 consecutive days and on day 4^th^ + high dose sildenafil (30 mg/kg); * Intergroup difference between group 1 and other groups. **^†^** Intergroup difference between group 2 and other groups. **^‡^** Intergroup difference between group 3 and other groups. **^§^** Intergroup difference between group 4 and other groups.

### 2.3. Pharmacokinetics of Sildenafil in High Dose *Epimedium sagittatum* Extract (2 g/kg/day, p.o.)

**Figure 3:**
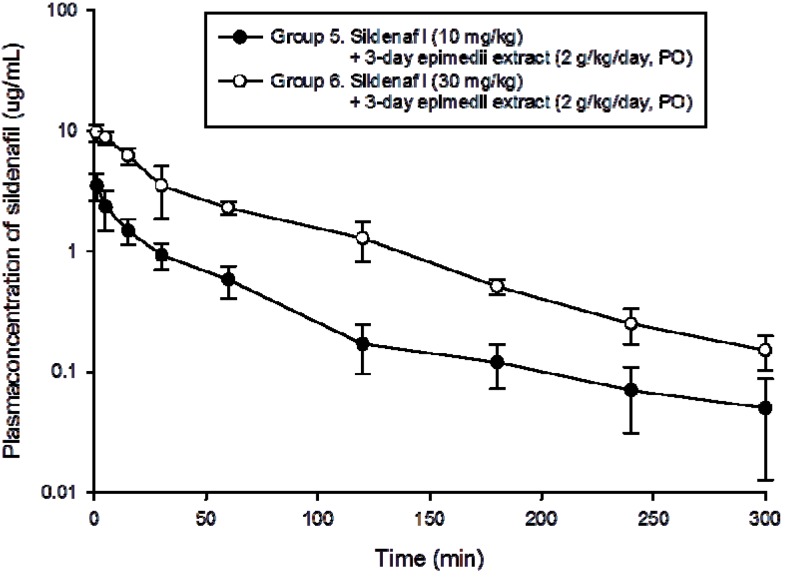
Plasma concentration-time profile of sildenafil in the blood of group 5: high dose *Epimedium sagittatum* (Sieb. et Zucc.) Maxim extract (2 g/kg/day) for 3 consecutive days and on day 4 + low dose sildenafil (10 mg/kg), group 6: high dose *Epimedium sagittatum* (Sieb. et Zucc.) Maxim extract (2 g/kg/day) for 3 consecutive days and on day 4 + high dose sildenafil (30 mg/kg). Data are presented as expressed as mean ± standard error of the mean (S.E.), n = 6 for each group.

Figure 3 showed the concentration-time profiles in the groups that received 2 g/kg *Epimedium sagittatum* extract pretreatment (groups 5 and 6). The half-life (t_1/2_) and Cl revealed no significant difference between group 5 and 6 (Table 4). The C_0_ data was significantly increased between groups 5 and 6 (3.3 *versus* 9.4, *p* < 0.001). The AUC data showed statistically significant differences between groups 5 and 6 (108 *versus* 440, *p* < 0.001), indicating a possible herb-drug interaction between sildenafil and *Epimedium sagittatum* extract.

**Table 4:** Pharmacokinetic parameters of sildenafil in group 5 and 6.

**Parameters**	**t_1/2_ (min)**	**C_0_ (μg/mL)**	**AUC (min μg/mL)**	**Cl (mL/min/kg)**	**V_ss_ (L/kg)**
Group 5	55 ± 17	3.3 ± 1.3	108 ± 34	111 ± 70	6540 ± 3760
Group 6	69 ± 17	9.4 ± 1.9 **	440 ± 106 **	73 ± 23	4790 ± 510

Group 5, high dose *Epimedium sagittatum* extract (2 g/kg/day) for 3 consecutive days and on day 4^th^ + low dose sildenafil (10 mg/kg); group 6, high dose *Epimedium sagittatum* extract (2 g/kg/day) for 3 consecutive days and on day 4^th^ + high dose sildenafil (30 mg/kg). Data are expressed as mean ± S. D. (n = 6). ** Intergroup difference between group 5 and other groups

### 2.4. Effects of *Epimedium sagittatum* Extract on the Pharmacokinetics of Sildenafil (10 mg/kg, i.v.)

The data were used to compare the effect of *Epimedium sagittatum* extract on the pharmacokinetics of sildenafil after normalization of sildenafil dose to 10 mg/kg in group 4 and group 6. For example, the dose-normalized (based on 10 mg/kg sildenafil) C_0_ for group 4 and group 6 was 3.9 ± 0.8 μg/mL and 3.1 ± 0.6 μg/mL, respectively. The dose-normalized (based on 10 mg/kg sildenafil) AUC for group 4 and group 6 was 178 ± 29 min μg/mL and 147 ± 35 min μg/mL, respectively. The half-life (t_1/2_) revealed no significant difference in all groups (Table 5). The C_0_ data showed statistically significant differences between groups 1 and 2 (5.6 *versus* 3.4, *p* = 0.014), which indicated the herbal drug interaction after immediate *Epimedium sagittatum* extract ingestion. The C_0_ data revealed statistically significant differences between groups 1 and 5 (5.6 *versus* 3.3, *p* = 0.01) and between groups 1 and 6 (5.6 *versus* 9.4, *p* = 0.004), while no statistically significant differences could be identified between groups 1 and 3 and between groups 1 and 4. These results were further supported by the statistically significant differences between group 3 and 5 (5.3 *versus* 3.3, *p* = 0.031) and between group 3 and 6 (5.3 *versus* 9.4, *p* = 0.015). This phenomenon might be resulted from the low dose of *Epimedium sagittatum* extract, which could not provide sufficient herbal drug interaction on the metabolism of intravenous sildenafil.

**Table 5:** Pharmacokinetic parameters of dose-normalized sildenafil in various groups.

**Parameters**	**t_1/2_ (min)**	**C_0_ (μg/mL)**	**AUC (min μg/mL)**	**Cl (mL/min/kg)**	**V_ss_ (L/kg)**
Group 1	80 ± 34	5.6 ± 0.9	348 ± 44	27 ± 3.6	2710 ± 627
Group 2	84 ± 17	3.4 ± 0.8 *	183 ± 15*	55 ± 5.0	5400 ± 588
Group 3	54 ± 3.8	5.3 ± 0.9 ^†^	296 ± 24 ^†^	33 ± 2.8	2460 ± 98
Group 4^a^	61 ± 8.5	3.9 ± 0.8	178 ± 29 *^,‡^	57 ± 8.9	3790 ± 552
Group 5	55 ± 17	3.3 ± 1.3 *^,‡^	108 ± 34 *^,†,‡,§^	111 ± 70 *^,‡^	6540 ± 3760 *^,‡^
Group 6^a^	69 ± 17	3.1 ± 0.6 *^,‡^	147 ± 35 *^,‡^	73 ± 23	4790 ± 510

Group 1, sildenafil alone (10 mg/kg); group 2, low dose *Epimedium sagittatum* extract (1 g/kg) 2 hr pretreated + low dose sildenafil (10 mg/kg); group 3, low dose *Epimedium sagittatum* extract (1 g/kg/day) for 3 consecutive days and on day 4^th^ + low dose sildenafil (10 mg/kg); group 4, low dose *Epimedium sagittatum* extract (1 g/kg/day) for 3 consecutive days and on day 4^th^ + high dose sildenafil (30 mg/kg); group 5, high dose *Epimedium sagittatum* extract (2 g/kg/day) for 3 consecutive days and on day 4^th^ + low dose sildenafil (10 mg/kg); group 6, high dose *Epimedium sagittatum* extract (2 g/kg/day) for 3 consecutive days and on day 4^th^ + high dose sildenafil (30 mg/kg). Data are expressed as mean ± S. D. (n = 6). ^a^C_0_ and AUC was normalized to the dose of 10 mg/kg when statistical analysis was performed. ^*^Intergroup difference between group 1 and other groups. **^†^** Intergroup difference between group 2 and other groups. **^‡^** Intergroup difference between group 3 and other groups. **^§^** Intergroup difference between group 4 and other groups.

The AUC data showed statistically significant differences between group 1 and other groups, except for group 3, which indicated the possible dose-dependent herbal drug interaction between *Epimedium sagittatum* extract and sildenafil. For groups that received 3 days *Epimedium sagittatum* extract (group 3, 4, 5, 6), dose-normalized AUC values showed statistically significant differences between group 3 and other groups, and between group 4 and 5, which indicated further support to the dose-dependent herbal drug interaction between *Epimedium sagittatum* extract and sildenafil. The Cl showed statistically significant differences between group 1 and group 5 (27 *versus* 111, *p* = 0.003) and between group 3 and group 5 (33 *versus* 111, *p* = 0.007). The V_ss_ data showed higher distribution of sildenafil between group 1 and group 5 (2710 *versus* 6540, *p* = 0.014) and between group 3 and group 5 (2460 *versus* 6540, *p* = 0.008).

### 2.5. Discussion

Phosphodiesterase 5 inhibitors are common medications for managing erectile dysfunction and *Epimedium sagittatum* is one of the common herbs used in Traditional Chinese Medicine for this purpose. We choose sildenafil and *Epimedium sagittatum* as our study targets to investigate the herb-drug interaction between Western and Traditional Chinese Medicines. To our knowledge, this is the first pharmacokinetic study of phosphodiesterase 5 inhibitors and the *Epimedium sagittatum* species.

In this study, dose-normalized C_0_ data showed statistically significant differences between group 1 (controlled group) and 2 (immediate *Epimedium sagittatum* extract group). Similar results were found between group 3 (low dose *Epimedium sagittatum* extract group) and 5 (high dose *Epimedium sagittatum* extract group), and between groups 3 and 6 (low dose *Epimedium sagittatum* extract group *versus* high dose *Epimedium sagittatum* extract group). We found that the higher the dosage of *Epimedium sagittatum* extract, the lower C_0_ was found in this study, which might provide further information regarding the herbal drug interaction between *Epimedium sagittatum* extract and sildenafil. In 2006, Shin *et al*. reported the pharmacokinetics of sildenafil in the blood and portal system in rats. They found dosage dependent phenomenon of sildenafil between 10 to 50 mg/kg in rats [25]. In order to conduct an in-depth analysis of herbal drug interaction between Epimedium sagittatum extract and sildenafil, dose normalization of sildenafil (from 30 mg/kg to 10 mg/kg) was performed in our study. We found that AUC of sildenafil showed statistically significant differences between group 1 and 2, which suggest the low dose *Epimedium sagittatum* extract might have an immediate impact on the metabolism of sildenafil. In group 1, 3, 4, 5 and 6, the effect of short-term *Epimedium sagittatum* extract on sildenafil was investigated. The AUC of sildenafil after dose normalization revealed statistically significant differences between group 1 and 4, between group 1 and 5 and between group 1 and 6, which might indicate herbal drug interaction between *Epimedium sagittatum* extract and sildenafil. With the increment of the dosage of *Epimedium sagittatum* extract, statistically significant differences between *Epimedium sagittatum* extract and sildenafil were found between groups 3 and 5, groups 3 and 6, and groups 4 and 5. There was no statistically significant difference found between groups 1 and 3. From those data, we suggested that *Epimedium sagittatu*m extract might have partial pharmacokinetic effect on sildenafil. Since sildenafil is mainly metabolized by hepatic enzymes [9,10], it is reasonable to hypothesize *Epimedium sagittatum* extract might have potential effects on liver enzymes or ATP-binding cassette-carriers, such as P-glycoprotein, multi-drug resistance protein 2 or breast cancer resistance protein [26,27].

There were some limitations to this study. First, we used *Epimedium sagittatum* extract rather than isolated herbal ingredients from *Epimedium sagittatum*. In fact, we tried to demonstrate the actual clinical scenario regarding the daily life of Chinese culture in this study. In Traditional Chinese Medicine, people take Herba Epimedii extract rather than the isolated herbal ingredients, such as icariin, epimedium B or epimedium C. To investigate the herbal drug interaction in this study, the *Epimedium sagittatum* extract was used to find the real effect between sildenafil and *Epimedium sagittatum* extract. Second, the pretreatment period of *Epimedium sagittatum* extract might have been too short to identify the possible herbal drug effect. In 2010, Shindel *et al*. reported the use of icariin, the main components of *Epimedium sagittatum*, could induce erectogenic and neurotrophic effects in castrated rats [28]. They indicated that continuous use low dose icariin for 28 days might significantly affect neurite growth of pelvic ganglion and increase intracavernous pressure in the penile tissues. However, the use of icariin differs from *Epimedium sagittatum* extract in our study, meaning the comparison between these two studies requires further investigation. Besides, the different pretreatment period (3 days *versus* 28 days) is another consideration in comparing the different data from these studies. Hence, the necessity to prolong the pretreatment period of *Epimedium sagittatum* extract deserves further investigation and will be conducted in upcoming research projects. Third, the dosage of *Epimedium sagittatum* extract treatment for rats in this study was high, and it is expected *Epimedium sagittatum* extract may have no significant effect on the pharmacokinetics of sildenafil at a normal daily ingested dose.

## 3. Experimental

### 3.1. Chemicals and Reagents

Sildenafil was obtained from Pfizer Pharmaceuticals (New York, NY, USA). Liquid chromatographic grade solvent and reagents were purchased from E. Merck (Darmstadt, Germany). Triplied deionized water was prepared by the Milli-Q system (Millipore, Bedford, MA, USA) and used for all preparations.

### 3.2. Preparation of *Epimedium sagittatum* (Sieb. et Zucc.) Maxim Extract

*Epimedium sagittatum* (Sieb. et Zucc.) Maxim was purchased from a local traditional Chinese medicine distributor (Taipei, Taiwan) and was verified by Ching-Song Shyu, director of Chia Huei Co., Inc., Taipei, Taiwan, based on comparisons of the external appearance of the leaves with the figures drawn in the literature [29]. A voucher specimen was deposited in the National Research Institute of Chinese Medicine, Taipei, Taiwan. *Epimedium sagittatum* (10 kg) was immersed in 160 liters of ethanol and extracted two times. A total of 320 liters *Epimedium sagittatum* extract were collected and filtered through layers of gauze. The residues were discarded and the filtrates were kept at −20 °C and frozen. The final weight of the *Epimedium sagittatum* extract was 1,076 g.

### 3.3. Liquid Chromatography

HPLC-UV instrumentation was performed with a Shimadzu chromatographic pump (LC-20AT), a DGU-20A5 degasser, an autosampler (SIL-20AC) and a photo-diode array detector (SPD-M20A) (Shimadzu, Kyoto, Japan). *Epimedium sagittatum* extract and sildenafil were separated using a Phenomenex Kinetex C_18_ column (100 × 2.10 mm, 2.6 µm, Torrance, CA, USA) and maintained in an oven (40 °C). The mobile phase was composed of acetonitrile/10 mM KH_2_PO_4_ + triethylamine (99.5:0.5, v/v, pH 6.0 adjusted with orthophosphoric acid) at the ratio 70:30 (v/v). The flow rate of the mobile phase was set at 0.4 mL/min. The injected sample volume was 10 μL and the UV detection wavelength was set at 220 nm. 

### 3.4. Plasma Preparation of Sildenafil Extraction

Plasma (100 μL) was alkalized with NaOH (1 mM, 20 μL) and extracted with ethyl acetate (500 μL) for liquid-liquid extraction. The samples were vortex-mixed for 5 min and centrifuged at 6000 × g for 10 min, and this process was repeated three times. The combined extraction solution was then evaporated to dryness. The residues were reconstituted in 100 μL mobile phase. The samples were vortex-mixed and centrifuged at 3200 × g for 10 min. The supernatants were filtered through 0.22 μm membrane filter prior to HPLC analysis. Calibration standards of plasma samples were prepared by adding known amounts of sildenafil into blank rat plasma to give a range of 0.05 to 10 μg/mL. The inter-day and intra-day assays of sildenafil in rat plasma were determined by quantitating six replicates using the HPLC method.

### 3.5. Study Protocol

The study protocol has been reviewed and approved by the Institutional Animal Care and Use Committee (IACUC, approval number 1010911) by the Institutional Animal Experimentation Committee of National Yang-Ming University, Taipei, Taiwan. Male Sprague-Dawley rats (6 weeks, 250 ± 20 g body weight) were purchased from the laboratory animal center of National Yang-Ming University. All experimental animals were housed in standard rat cages on a 12-hour light/dark cycle under institutional guidelines and had free access to food and water throughout the entire experiment until 12 hours before the intravenous sildenafil administration. This study was conducted over a 4-day period and the rats were divided into six groups: group (1) sildenafil alone (10 mg/kg), (2) low dose *Epimedium sagittatum* extract (1 g/kg) 2 h pretreated + low dose sildenafil (10 mg/kg), (3) low dose *Epimedium sagittatum* extract (1 g/kg/day) for 3 consecutive days and on day 4^th^ + low dose sildenafil (10 mg/kg), (4) low dose *Epimedium sagittatum* extract (1 g/kg/day) for 3 consecutive days and on day 4^th^ + high dose sildenafil (30 mg/kg), (5) high dose *Epimedium sagittatum* extract (2 g/kg/day) for 3 consecutive days and on day 4^th^ + low dose sildenafil (10 mg/kg), (6) high dose *Epimedium sagittatum* extract (2 g/kg/day) for 3 consecutive days and on day 4^th^ + high dose sildenafil (30 mg/kg). The dosage regimens of *Epimedium sagittatum* extract and sildenafil are listed in Table 2. On day 4^th^, the rats were initially anesthetized with urethane (1 g/mL) and α-chloralose (0.1 g/mL) at a dosage of 1 mL/kg intraperitoneally anesthetized and remained anesthetized during the experiment. *Epimedium sagittatum* extract (1 g/mL) was suspended in 2% carboxymethyl cellulose (1 g/mL) for drug administration. Group 1 received vehicle (2% carboxymethyl cellulose, 1 g/mL) for 3 days as the control group. 0.5 mL sildenafil (10/30 mg/kg) was infused via left femoral vein of the rat for 5 s. 0.2 mL blood sample was collected from right external jugular vein at 0, 1, 5, 15, 30, 60, 120, 180, 240, 300 min after sildenafil administration. At the end of the experiment, the rats were euthanized with an overdose of carbon dioxide while still under anesthesia.

### 3.6. Surgical Procedures

The rat was put in supine position and the four extremities were fixed with elastic bandages. One 0.8 cm incision was made at left femoral canal and dissected layer by layer till identification of left femoral vein. Vascular cannulation was performed for intravenous sildenafil injection. Another 0.8 cm incision was made at right neck and dissected layer by layer till identification of right external jugular vein. Vascular cannulation was performed for sequential blood sampling. 

### 3.7. Data Analysis

The WinNonlin Standard Edition (Version 1.1, Scientific Consulting Inc., Apex, NC, USA) was used to calculate pharmacokinetic data. An IV-bolus input non-compartmental model was used to find the blood pharmacokinetic parameters, including elimination half life (t_1/2_), maximum concentration of drug (C_0_), area under the concentration-time curve (AUC), clearance (Cl) and the apparent volume of distribution at steady state (V_ss_). The results were presented as mean ± standard deviation. Statistical analysis was performed by student *t*-test and one-way ANOVA with post-Hoc test using SPSS programs (version 18.0, Chicago, IL, USA), while a *p* value < 0.05 was considered statistically significant. 

## 4. Conclusions

Based on a pharmacokinetics perspective, there was a significant herb-drug interaction between *Epimedium sagittatum* extract and sildenafil. Patients who considered integrated use of *Epimedium sagittatum* extract and sildenafil might be advised to prevent possible therapeutic failure due to enhanced elimination of sildenafil. From this study, we hope to provide more information on integrating eastern herbal medicine and western medications for managing erectile dysfunction in clinical practice. 

## References

[1] Chen K.K., Chiang H.S., Jiann B.P., Lin J.S., Liu W.J., Wu C.J., Hsieh J.T., Wang C.J., Hwang T.I., Lee S.S. (2004). Prevalence of erectile dysfunction and impacts on sexual activity and self-reported intercourse satisfaction in men older than 40 years in Taiwan. Int. J. Impot. Res..

[2] Jønler M., Moon T., Brannan W., Stone N.N., Heisey D., Bruskewitz R.C. (1995). The effect of age, Ethnicity and geographical location on impotence and quality of life. Br. J. Urol..

[3] Althof S.E. (2002). Quality of life and erectile dysfunction. Urology.

[4] Esposito K., Giugliano F., Di Palo C., Giugliano G., Marfella R., D’Andrea F., D’Armiento M., Giugliano D. (2004). Effect of lifestyle changes on erectile dysfunction in obese men: a randomized controlled trial. JAMA.

[5] McVary K.T. (2007). Clinical practice. Erectile dysfunction. N. Engl. J. Med..

[6] Tsertsvadze A., Fink H.A., Yazdi F., MacDonald R., Bella A.J., Ansari M.T., Garritty C., Soares-Weiser K., Daniel R., Sampson M. (2009). Oral phosphodiesterase-5 inhibitors and hormonal treatments for erectile dysfunction: a systematic review and meta-analysis. Ann. Intern. Med..

[7] Eardley I., Donatucci C., Corbin J., El-Meliegy A., Hatzimouratidis K., McVary K., Munarriz R., Lee S.W. (2010). Pharmacotherapy for erectile dysfunction. J. Sex. Med..

[8] Nichols D.J., Muirhead G.J., Harness J.A. (2002). Pharmacokinetics of sildenafil after single oral doses in healthy male subjects: Absolute bioavailability, food effects and dose proportionality. Br. J. Clin. Pharmacol..

[9] Walker D.K., Ackland M.J., James G.C., Muirhead G.J., Rance D.J., Wastall P., Wright P.A. (1999). Pharmacokinetics and metabolism of sildenafil in mouse, rat, rabbit, dog and man. Xenobiotica.

[10] Hyland R., Roe E.G., Jones B.C., Smith D.A. (2001). Identification of the cytochrome P450 enzymes involved in the N-demethylation of sildenafil. Br. J. Clin. Pharmacol..

[11] Xu W., He S. (2005). Species and geographic distribution of large-flowered taxa of Epimedium in China. Zhong Yao Cai.

[12] Wang S., Zheng Z., Weng Y., Yu Y., Zhang D., Fan W., Dai R., Hu Z. (2004). Angiogenesis and anti-angiogenesis activity of Chinese medicineal herbal extracts. Life Sci..

[13] Liu K.H., Kim M.J., Jeon B.H., Shon J.H., Cha I.J., Cho K.H., Lee S.S., Shin J.G. (2006). Inhibition of human cytochrome P450 isoforms and NADPH-CYP reductase *in vitro* by 15 herbal medicines, including Epimedii herba. J. Clin. Pharm. Ther..

[14] Liu B., Zhang H., Xu C., Yang G., Tao J., Huang J., Wu J., Duan X., Cao Y., Dong J. (1375). Neuroprotective effects of icariin on corticosterone-induced apoptosis in primary cultured rat hippocampal neurons. Brain Res..

[15] Chiu J.H., Chen K.K., Chien T.M., Chiou W.F., Chen C.C., Wang J.Y., Lui W.Y., Wu C.W. (2006). Epimedium brevicornum Maxim extract relaxes rabbit corpus cavernosum through multitargets on nitric oxide/cyclic guanosine monophosphate signaling pathway. Int. J. Impot. Res..

[16] Makarova M.N., Pozharitskaya O.N., Shikov A.N., Tesakova S.V., Makarov V.G., Tikhonov V.P. (2007). Effect of lipid-based suspension of Epimedium koreanum Nakai extract on sexual behavior in rats. J. Ethnopharmacol..

[17] Liu T., Xin H., Li W.R., Zhou F., Li G.Y., Gong Y.Q., Gao Z.Z., Qin X.C., Cui W.S., Shindel A.W. (2011). Effects of icariin on improving erectile function in streptozotocin-induced diabetic rats. J. Sex. Med..

[18] Ma H., He X., Yang Y., Li M., Hao D., Jia Z. (2011). The genus Epimedium: an ethnopharmacological and phytochemical review. J. Ethnopharmacol..

[19] Cheng S., Qiu F., Wang S., He J. (2007). HPLC analysis and pharmacokinetics of icariin in rats. J. Sep. Sci..

[20] Shen P., Wong S.P., Li J., Yong E.L. (2009). Simple and sensitive liquid chromatography-tandem mass spectrometry assay for simultaneous measurement of five Epimedium prenylflavonoids in rat sera. J. Chromatogr. B.

[21] Hu D.D., Yao H.J., Gu L., Wang S.P., Liu G.L. (2008). Effect of total flavonoids of epimedium on liver microsomal CYP1A2, CYP3A4 and CYP2E1 activities in rats. JCPS.

[22] Wu Y.T., Lin C.W., Lin L.C., Chiu A.W., Chen K.K., Tsai T.H. (2010). Analysis of biliary excretion of icariin in rats. J. Agric. Food Chem..

[23] Katz B.F., Stember D.S., Nagler H.M. (2011). Sexual medicine disparities between Asia and North America: commentary on male sexual dysfunction in Asia. Asian J. Androl..

[24] Zhang E.Y. (2007). Switching between traditional chinese medicine and viagra: cosmopolitanism and medical pluralism today. Med. Anthropol..

[25] Shin H.S., Bae S.K., Lee M.G. (2006). Pharmacokinetics ofsildenafilafter intravenous and oral administration in rats: hepatic and intestinal first-pass effects. Int. J. Pharm..

[26] Kim R.B. (2002). Transporters and xenobiotic disposition. Toxicology.

[27] Kim R.B. (2002). Drugs as P-glycoprotein substrates, Inhibitors, and inducers. Drug Metab. Rev..

[28] Shindel A.W., Xin Z.C., Lin G., Fandel T.M., Huang Y.C., Banie L., Breyer B.N., Garcia M.M., Lin C.S., Lue T.F. (2010). Erectogenic and neurotrophic effects of icariin, a purified extract of horny goat weed (Epimedium spp.) *in vitro* and *in vivo*. J. Sex. Med..

[29] Zhang C., Chen D.C., Lin H.R., Lu H.W. (1999). Zhong Kuo Zhong Yao Tsai Chen Wei Chien Pei Tu Dian” (Verified Illustrations of Chinese Materia Medica).

